# Supplementary figures and images for: Estrogen receptor alpha mediated repression of PRICKLE1 destabilizes REST and promotes uterine fibroid pathogenesis
Source: bioRxiv. 2024 Sep 9:2024.09.09.612036. Preprint. [Version 1] doi: 10.1101/2024.09.09.612036 (PMC11419101; doi:10.1101/2024.09.09.612036)

Supplemental Figures

Fig. S1

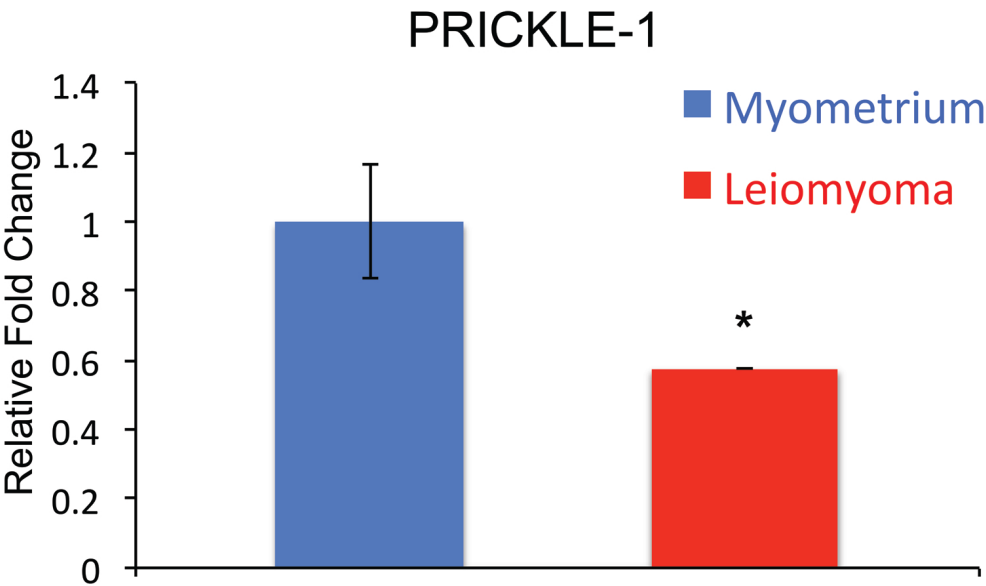

**Myometrium**

**Leiomyoma**

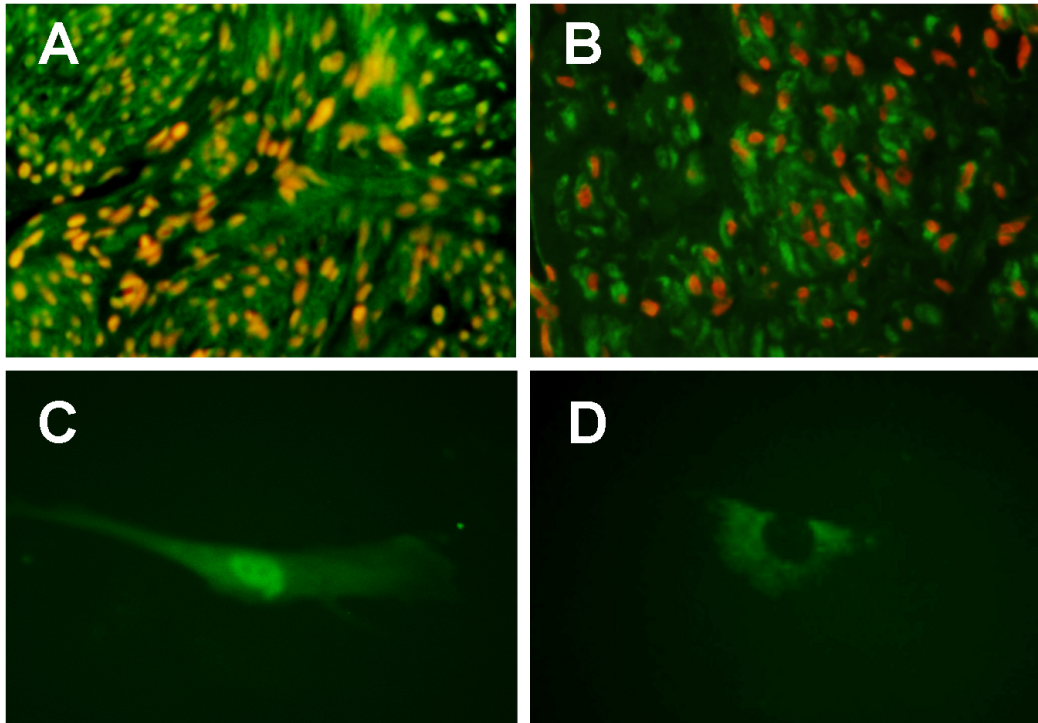

**Fig. S2**

Fig. S3

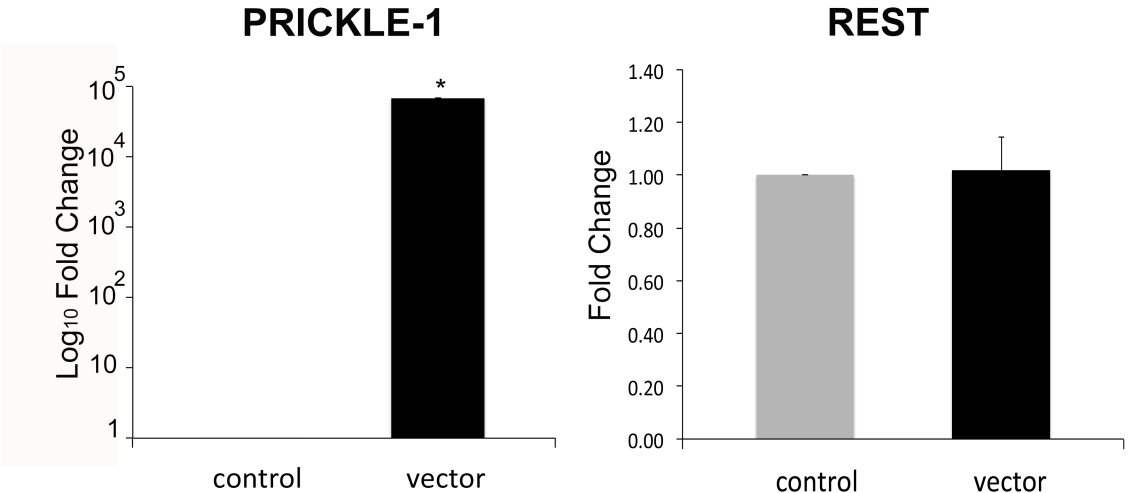

**Fig. S4**

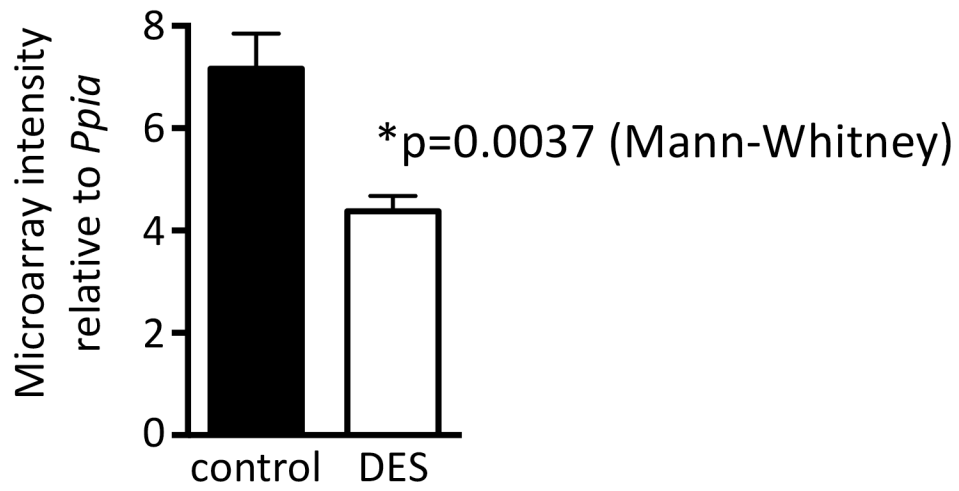

Fig. S5

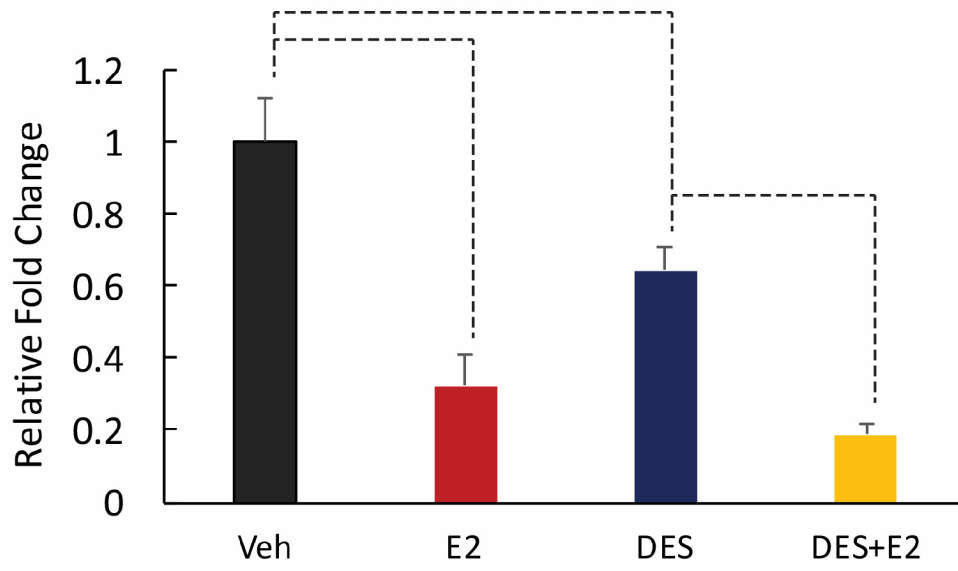

**Fig. S6**

**A**

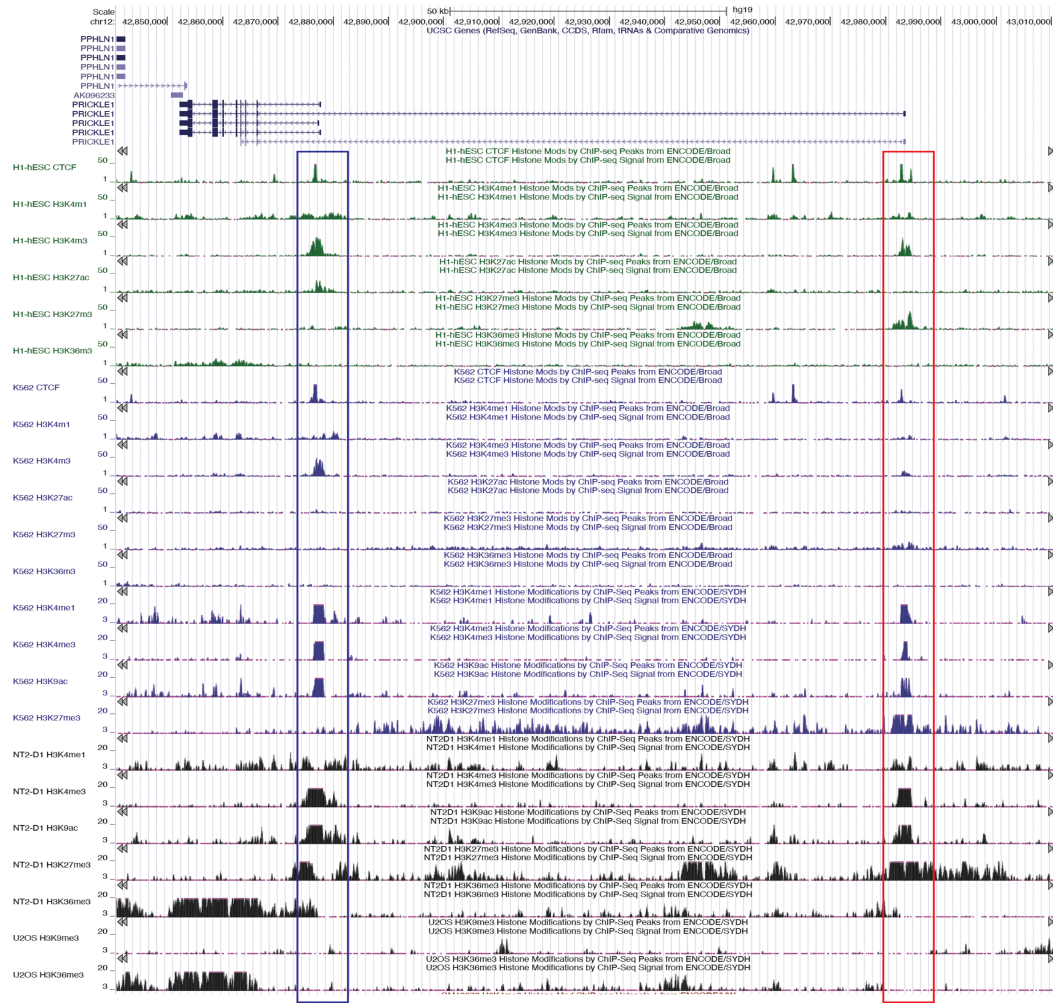

**B**

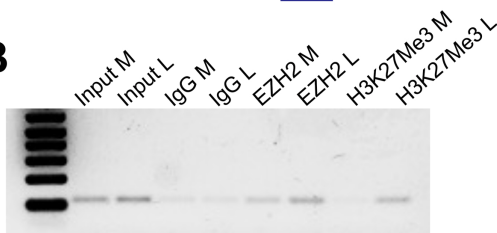

**C**

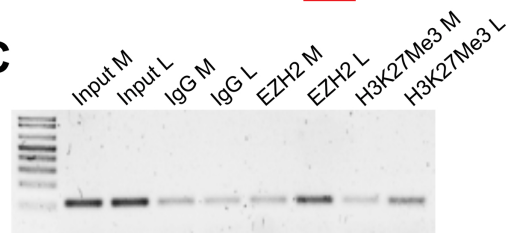

Fig. S 7

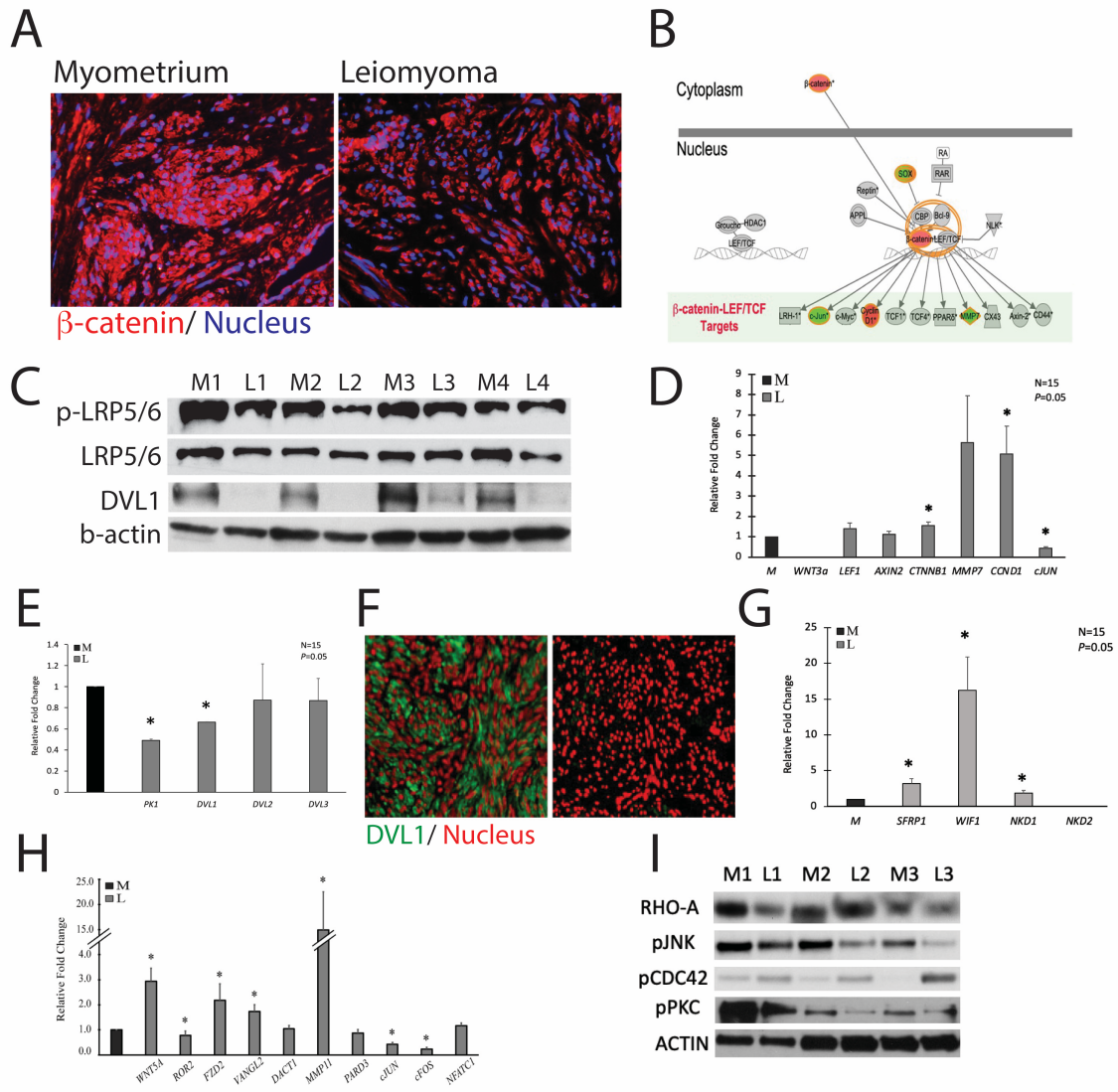

Supplement: Supplement 1 — Fig. S1. PRICKLE-1 mRNA expression is decreased in uterine leiomyomas. Gene expression analysis of PRICKLE1 in myometrium and leiomyoma tissue samples (12 pairs). * P < 0.05 Fig. S2. REST localization is primarily cytoplasmic in leiomyomas. (A & B) Immunofluorescence analysis of REST (green) in myometrial and leiomyoma tissue samples. (C &D). Immunofluorescence analysis of GFP-REST(green) transfected MSMCs and LSMCs. Nuclei were stained with EthD-2 (red). Fig. S3. PRICKLE1 regulates REST at the protein level. Gene expression analysis of PRICKLE1 and REST in LSMCs overexpressing FLAG-PRICKLE-1. Fig. S4. Neonatal (PND1-5) DES exposure suppresses Prickle1 expression in the mouse uterus. Relative mRNA levels from Affymetrics gene expression arrays Fig. S5. Neonatal exposure to DES augments the suppression of Prickle1 by 17β – estradiol. TaqMan qRT-PCR assay showing relative Prickle1 mRNA expression in mice treated neonatally (PND1-5) with vehicle or DES, ovariectomized at 8 weeks of age and then treated with vehicle or 17β - estradiol. Effect of estradiol on Prickle1 mRNA expression in control mice treated neonatally (PND1-5) with vehicle (samples; veh, E2), effect of estradiol on Prickle1 mRNA expression in adult ovariectomized mice which were treated neonatally (PND1-5) with DES (samples; DES, DES + E2). Dotted lines indicate P< 0.05 Fig. S6. EZH2 mediated repression of PRICKLE1 in leiomyomas. A. UCSC genome browser data showing H3K27Me3 peaks near the major TSS sequences of PRICKLE1. B, C, representative ChIP PCR from patients showing association of EZH2 to the distal start site (red box in A) and increased H3K27 methylation in UL. Fig. S7. Disruption of WNT/ planar cell polarity pathway in leiomyomas. A. Immunofluorescence staining of β-catenin showing predominant cytoplasmic localization in UL tissue. B. Analysis of gene expression dataset GSE13319 showing downregulation (green) or upregulation (red) of TCF/LEF targets. C. Western blot analysis showing the absence [file media-1.pdf]
